# Relationships among Traits of Aerobic and Anaerobic Swimming Performance in Individual European Sea Bass *Dicentrarchus* *labrax*

**DOI:** 10.1371/journal.pone.0072815

**Published:** 2013-09-03

**Authors:** Stefano Marras, Shaun S. Killen, Paolo Domenici, Guy Claireaux, David J. McKenzie

**Affiliations:** 1 Institut des Sciences de l'Evolution de Montpellier, UMR5554, Université de Montpellier 2, Montpellier, France; 2 IAMC-CNR, Località Sa Mardini, Torregrande, Oristano, Italy; 3 Université de Bretagne Occidentale, LEMAR UMR6539, PFOM-ARN, Centre Ifremer de Brest, Plouzane, France; University of Hamburg, Germany

## Abstract

Teleost fishes exhibit wide and temporally stable inter-individual variation in a suite of aerobic and anaerobic locomotor traits. One mechanism that could allow such variation to persist within populations is the presence of tradeoffs between aerobic and anaerobic performance, such that individuals with a high capacity for one type of performance have a reduced capacity for the other. We investigated this possibility in European seabass *Dicentrarchus* *labrax* , each measured for a battery of indicators of maximum locomotor performance. Aerobic traits comprised active metabolic rate, aerobic scope for activity, maximum aerobic swimming speed, and stride length, using a constant acceleration test. Anaerobic traits comprised maximum speed during an escape response, maximum sprint speed, and maximum anaerobic burst speed during constant acceleration. The data provided evidence of significant variation in performance among individuals, but there was no evidence of any trade-offs among any traits of aerobic versus anaerobic swimming performance. Furthermore, the anaerobic traits were not correlated significantly among each other, despite relying on the same muscular structures. Thus, the variation observed may reflect trade-offs with other morphological, physiological or behavioural traits.

## Introduction

Consistent inter-individual variation in locomotor performance, such as aerobic endurance capacity, or anaerobic sprint/burst capacity, has been described in numerous animal taxa [1–6]. Locomotor performance is widely assumed to contribute to fitness in animals, so there has been interest in exploring how individuals with relatively poor performance can persist in populations. One major focus, in particular in vertebrates, has been on the existence of physiological trade-offs between performance traits, such that poor performance in one trait is associated with increased performance in another. In tetrapod, for instance, skeletal muscles contain a mixture of aerobic and anaerobic fibers potentially resulting in a trade-offs between aerobic (endurance) and anaerobic (sprint/burst) performance as increasing the proportion of one type of fibre can only occur at the expense of the others. Whereas some studies have found evidence of such a trade-off in tetrapods [7–12], others have not [13–21].

In fishes, aerobic and anaerobic swimming are powered by distinct muscle groups [22–24]. Slow-twitch oxidative “red” muscles sustain steady-state aerobic swimming at slow to moderate speeds. In theory these muscles can be supported indefinitely by aerobic metabolism, oxygen and nutrients being continuously supplied by the cardiovascular and respiratory systems. Examples of steady-state aerobic swimming are migrations or maintaining station against currents [25–27]. Fast-twitch glycolytic “white” muscles are used for brief periods of anaerobic swimming at high speeds, fuelled by endogenous substrates that must be replenished during a recovery period. Examples of high speed anaerobic swimming are escape responses, brief sprints or more extended bursts during predator–prey encounters [25,26,28]. Intrinsic variation in traits of swimming performance in fishes has been reported in a number of species, for example for largely aerobic traits such as “critical” swimming speed (*U*_crit_) measured in a swim tunnel, or purely anaerobic traits such as escape swimming speed (*U*_escape_) and sprint swimming speed (*U*_sprint_) measured with high speed video or a sprint chamber, respectively [3–5,29–31]. Theoretical arguments have been put forward in support of a potential trade-off in fishes [26,32] but few studies have actually investigated it. Previous work [10] reported a trade-off between *U*_crit_ and anaerobic burst swimming speed in the Atlantic cod *Gadus morhua*, and a positive correlation between *U*_crit_ and *U*_sprint_. By contrast, however, Claireaux and colleagues [3] and Seebacher and Walter [33] found no correlations between *U*_crit_ and *U*_sprint_ in the European sea bass *Dicentrarchus* *labrax* or in the zebrafish *Danio rerio*, respectively. Work on the guppy *Poecilia* *reticulata* [30] found no correlations between *U*_crit_, *U*_escape_ and the maximum bursting speed achieved during a constant acceleration test (CAT) in a swim tunnel (*U*_burst_).

The European sea bass *Dicentrarchus* *labrax* is a body caudal fin swimmer; an active predator that captures by pursuit. It undertakes extensive seasonal migrations between inshore feeding grounds and offshore spawning areas [34,35]. As a consequence, it can perform well in both sustained aerobic and brief anaerobic swimming modes [3,4,36–38] and is an interesting model to investigate the existence of performance trade-offs.

Because aerobic swimming relies on the integrated function of the cardiorespiratory system and red muscles (i.e. uptake, delivery and usage of oxygen), there is no single test that is currently accepted as the defining measure of maximum aerobic performance. Using juvenile sea bass, we measured a range of traits associated with cardiorespiratory and aerobic swimming performance. These included routine metabolic rate (RMR) and active metabolic rate (AMR), which allowed derivation of aerobic scope for activity (AS). Maximum aerobic speed was estimated as the speed of gait transition from steady rhythmic aerobic to burst-and-coast anaerobic swimming (*U*_gt_) during a CAT in a swim tunnel [4], while average and maximum aerobic stride length were measured as the distance swum per aerobic tail beat. We also measured the main anaerobic swimming performance traits sustained by white muscles, namely *U*_escape_ and *U*_sprint_, using high speed video and a sprint chamber, respectively. The maximum swimming speed achieved during a CAT (*U*_CAT_) was also measured [3,4], as well as the rate of post-exercise recovery following CAT. We then explored correlations among these various traits, with the hypothesis that traits which use different muscular systems would be negatively correlated, whereas those that use the same system would be positively correlated.

## Materials and Methods

### Ethics Statement

The fishes were held, and the non-lethal experiments were conducted, in strict accordance with the laws governing animal experimentation in France. The experiments were performed by the holder of an animal experimentation licence issued by the University of Montpellier (D.J.M., Formation Experimentation Animale niveau 1, numéro d’agrément de la formation I-UnivMontp-F1-06). The S.M.E.L. facility, where the fish were held and the experiments performed, is recognised by the University of Montpellier as a certified facility for fish rearing and ecophysiological experimentation.

### Animals

Juvenile European sea bass were obtained from a local fish farm (Salses le Chateau, Languedoc Roussillon, France; 42°49´ N; 2°57´ E). They were first-generation fish raised in aquaculture from fertilized eggs obtained from wild broodstock captured in the western Mediterranean, and were reared in large concrete raceways. Fish were transported to the Station Méditerranéenne de l’Environnement Littoral in Sète, where they were transferred to a square tank (0.8 m^2^) supplied with re-circulated natural seawater maintained at constant temperature (20±0.3°C) and salinity (35.1±0.2‰), under a natural photoperiod. Six months later, when the initial holding tank was not large enough to maintain the growing fish, they were transferred to a 3 m^2^ rectangular tank and kept undisturbed under the same conditions for 2 months before the beginning of the experimentation. Fish were fed ad-lib four times a week with commercial pellets (Aphytec, Mèze, France). Feeding was interrupted at least 24 h before experimentation. At experimentation, fish measured 21 ±1.2 cm fork length and 113 ±21 g mass (mean ±S.D.).

### Experimental protocol

All traits were measured on every individual. The experiment started by measuring *U*_escape_. Fish were transferred to a video arena, and left undisturbed for 60 minutes before testing (see below for details). After this, the fish was removed from the fast-start arena and transferred to a sprint chamber, and left undisturbed for 60 minutes before testing for *U*_sprint_ (see below for details). At the end of the sprint test the fish was transferred to a Steffensen-type swim tunnel, and left undisturbed for 60 minutes before measuring *U*_gt_, stride length and *U*_burst_ in a CAT (see below for details). At the end of the CAT, oxygen consumption was measured for a period of 22 h, to estimate AMR, RMR and AS (see below for details). All experiments were conducted at 20 °C and fish transfers between experimental set-up and rearing tank were always without air exposure.

### Escape performance

The experimental set-up was as described in [5], comprising a circular tank (100 cm diameter x 80 cm depth and 25 cm water depth), supplied with re-circulating seawater at 20 °C. The escape response of the fish was induced by mechanical stimulation. The stimulus was a PVC cylinder with a tapered point and an iron bolt at the opposite end (10 cm height, 2 cm diameter and weighing 35 g). The stimulus was released by an electromagnet from a height of 150 cm above the water surface. To prevent visual stimulation before contact with the water surface, the stimulus was released into a vertical PVC tube (15 cm of diameter) ending 0.5 cm before the water surface [39]. Floodlighting was supplied by two 250W spotlights and the whole setup was covered by a black tarpaulin, to screen the fish from visual disturbance. A high speed camera (Red Lake Motion Scope) was positioned above the experimental tank. It was connected to a PC by a Pinnacle video acquisition system (Avid Technology, Inc., NY, USA) and recorded the escape response at 250 Hz. The camera was triggered to record from 1 s before the stimulation to 3 s after the stimulation. The fish was tested three times with a 30 min interval between stimulations. The maximum escape speed was evaluated within a fixed time of 60ms, which approximately corresponded to the mean duration of stage 1 and 2. The individual *U*_escape_ was taken to be the fastest speed achieved in the three tests [5].

### Sprint performance

The sprint performance chamber was as described in [29]. Dimensions of the raceway were 2.00 m (length), 0.25 m (width) and 0.30 m (height). Light-emitting laser diodes (OnPoint Lasers, Inc., Eden Prairie, Minnesota, and Selectronic, Lille, France) with a power output of 5 mW, a wavelength of 645–670 nm, and a beam width of 1.1 mm were placed at intervals of 0, 0.02, 0.02, 0.04, 0.08, 0.16, 0.25, and 0.50 m from the point at which a fish would begin its sprint. The lasers were placed in front of clear glass windows on one side of the raceway. The laser beam was detected on the opposite side of the chamber by eight arrays of Photodarlington detectors (Honeywell International, Inc., Morristown, New Jersey). When activated by light, the Photodarlington detector array puts out a 5-V signal to one of eight inputs on a Biopac MP150 data acquisition board (Biopac systems, Inc, Goleta, CA, USA). Data were assimilated with AcqKnowledge V.3.7 software (Biopac systems, Inc, Goleta, CA, USA), while velocity was automatically calculated from the times of breakage of subsequent laser beams and the distance between detector arrays utilizing Labview software (National Instruments Corporation, Austin, Texas). A trial began by observing that the fish was oriented in a suitable position, arming the computer, and gently grasping (or attempting to grasp) the fish’s caudal peduncle. The fish would then burst down the raceway, triggering the photocell circuits such that the time elapsed between consecutive beam breakages was recorded. Each fish was sprinted between four and six times consecutively, with 5 min between subsequent sprint trials. Individual *U*_sprint_ was taken as the fastest speed achieved in the successive trials [3].

### Constant acceleration test

The CAT was performed in a 30-l Steffensen-type swim tunnel (Loligo Systems, Tjele, Denmark) thermoregulated at 20 ± 0.5°C. The working section of the tunnel was 55 cm in length, 14 cm in width and 14 cm in height. A PVC honeycomb grid and deflectors were placed in the recirculation loop to promote rectilinear flow and uniform velocity profiles. Water flow was produced by a variable-speed electrical motor and propeller. The water speed to motor voltage output relationship was established by measuring flow with a flow rate sensor (Vernier software and technology, Beaverton, OR, USA) in the middle of the swimming section and calculating the best-fit line by the method of least squares. Swimming speeds were corrected for maximum solid blocking effects [40,41]. During the test, water velocity was increased steadily by 10 cm s^-1^ min^-1^ according with previous work on the same species [4].

A Sony Mini DV camera (25 frames s^-1^) placed over the respirometer chamber was used to record fish swimming patterns during the test. Video analysis of the steady phase of swimming at *U*_gt_ was used to calculate average and maximum aerobic stride lengths; the distance covered by a fish per tail beat, where tail beat is defined as a complete oscillation of the tail (Hz [42]). Tail beat frequency was measured at four different water speeds (i.e. 30, 40, 50 cm s^-1^ and at *U*_gt_, when the fish was swimming in a steady position relative to the back of the tunnel. The water velocity in m s^-1^ divided by the tail beat frequency in Hz, as a fraction of body length (BL), was the stride length in BL. The average stride length was obtained by averaging the three stride lengths measured at fixed speeds, while maximum stride length was measured 30 s before reaching U_gt_. The U_gt_ was identified as the transition from steady (aerobic) swimming with regular rhythmic tailbeats to intermittent burst and coast swimming, and was taken as a measure of maximum aerobic speed [4].

Beyond *U*_gt_ the animal switched gait to intermittent bursts of high-frequency tail beats [4], which indicated that it was recruiting anaerobic fast-twitch glycolytic muscle fibers [26]. Eventually, as speed was increased, the fish fell back against the retaining grid at the posterior end of the swim-tunnel and was unable to resume swimming. This water velocity was considered to be U_CAT_, the maximum speed attainable by the fish using its highest anaerobic power output.

The swimming tunnel was equipped with an oxygen meter (Fibox 3-trace v3, PreSens Precision Sensing GmbH, Regensburg, Germany). Immediately at the end of the CAT, metabolic rate was measured as oxygen consumption (MO_2_, in mg kg^-1^ h^-1^) using intermittent closed respirometry [43], for 15 min every 30 min, as described by 44, for 24 h. The AMR was taken as the highest MO_2_ measured immediately after exhaustion, RMR calculated as the mean of the last four oxygen uptake measurements, AS as AMR minus RMR. Finally, recovery time from exhaustion was assessed as the time (h) required for oxygen uptake to return from AMR to RMR [4,45].

### Data analysis and statistics

Variables were tested for normality (K–S test) and then correlations between variables were assessed with Pearson product-moment correlations. In the case of variables related with more than one other variable, step-wise multiple linear regressions were used to assess which of the independent variables was able to predict the dependent variable. A sequential Bonferroni procedure was applied to correct for multiple comparisons. The coefficients of variation (CV = SD/mean) were used to assess the extent of variation in each of the measured traits. Repeatability of *U*_escape_ and *U*_sprint_ was tested using the intraclass correlation coefficient (ICC), the ratio of variance among individuals to the total variance (among + within), calculated from the mean square terms of the ANOVA [46,47]. A one-way repeated measure ANOVA was used to assess differences among maximum swimming speeds achieved using different types of swimming tests and a paired T-test was used to determine differences between average and maximum stride length. Values are given as mean ± SD. Statistical analyses were performed using SigmaStat 3.1 (Systat Software) and significance was accepted at P<0.05.

## Results

A total of 35 fish were tested (Total length 21 ±1.2 cm, 113 ±21 g mass, at the time of the experimentation). No relationship was found between the measured traits and individual’s length or mass (Linear regression; P>0.05). However, a high degree of inter-individual variation was found with the coefficient of variation (CV; %) ranging from 38.4% (time for recovery following CAT), to 13.7% (RMR; see Table 1).

**Table 1:** Coefficients of variation of 9 variables measured in European sea bass.

	**CV (%)**
*U*_escape_	18.7
*U*_sprint_	14.8
*U*_CAT_	23.5
*U*_gt_	17.1
Stride length	14.6
AMR	17.5
RMR	13.7
Aerobic scope	34.5
Recovery time	38.4

### Comparisons between aerobic and anaerobic traits

**Figure 1:**
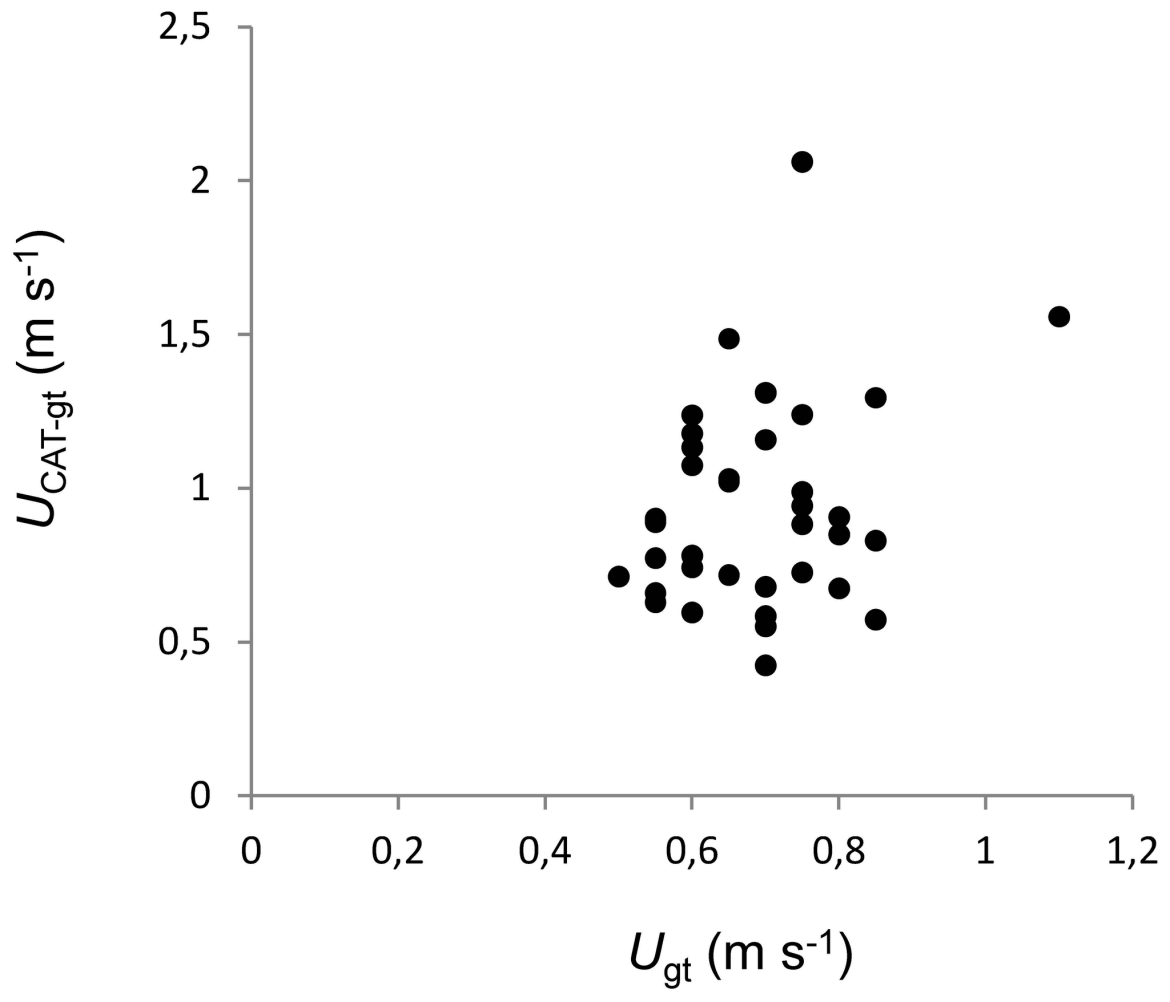
Relationship between aerobic gait transition speed (*U*_gt_) and values of U_CAT_ beyond *U*_gt_ (*U*_CAT-gt_).

**Figure 2:**
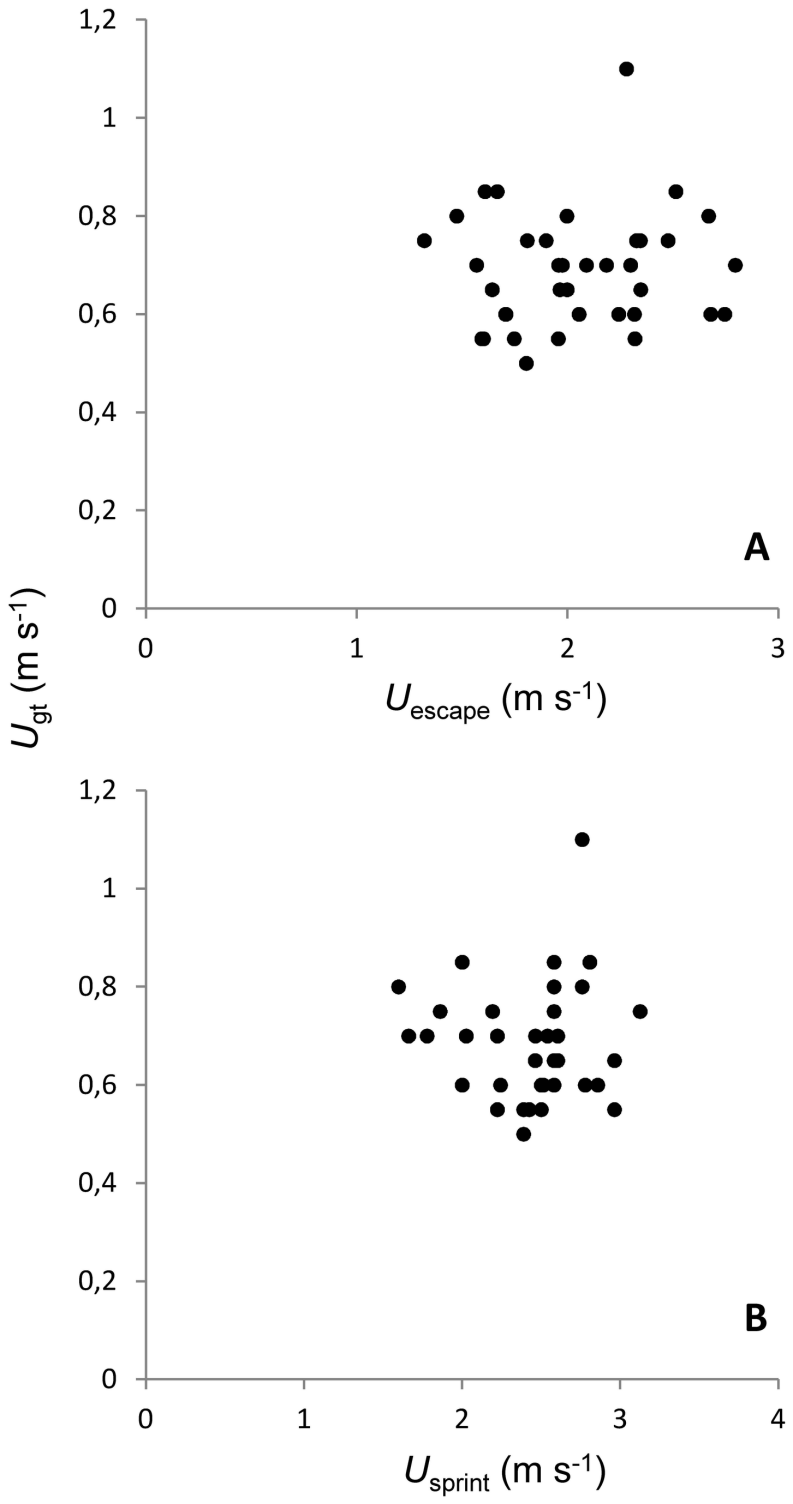
Relationship between (A) *U*_gt_ and *U*_escape_ and (B) between *U*_gt_ and *U*_sprint_.

*U*_gt_ and *U*_CAT_ were positively related (P<0.01). However, this correlation was expected since both measurements derived from the same test. To eliminate this autocorrelation the anaerobic component of U_CAT_ was calculated by subtracting *U*_gt_ from *U*_CAT__._ No correlation was found between the aerobic (U_gt_) and anaerobic components (*U*_CAT-gt_) of individuals performance during the constant acceleration test [(*U*_CAT_-_gt_)-*U*_gt_, P>0.05, Figure 1)]. Neither *U*_escape_ nor *U*_sprint_ were correlated with any aerobic traits (all P>0.05, Figure 2A,B, Table 2). *U*_CAT_-_gt_ was, however, positively correlated with both average and maximum stride length (all P<0.05, Table 2).

**Table 2:** Pearson correlations for the four swimming performance traits, stride length, metabolic rates and recovery time after exhaustion.

	***U*_sprint_**	***U*_CAT_**	***U*_gt_**	**Stride length**	**AMR**	**RMR**	**Aerobic scope**	**Recovery time**
***U*_escape_**	-0.126P=0.457	-0.173P=0.306	0.083P=0.626	0.082P=0.639	-0.073P=0.673	-0.119P=0.495	-0.027P=0.867	0.215P=0.216
***U*_sprint_**		0.165P=0.328	-0.002P=0.992	0.004P=0.998	0.245P=0.156	-0.144P=0.411	0.289P=0.092	0.235P=0.174
***U*_CAT_**			0.542P<0.001	0.663P<0.001	0.014P=0.937	-0.285P=0.097	0.118P=0.500	0.056P=0.748
***U*_gt_**				0.566P<0.001	-0.037P=0.831	-0.372P=0.028	0.101P=0.565	0.051P=0.772
**Stride length**					-0.033P=0.850	-0.229P=0.186	0.052P=0.767	-0.046P=0.797
**AMR**						0.090P=0.607	0.931P<0.001	0.164P=0.347
**RMR**							-0.280P=0.043	-0.231P=0.182
**Aerobic scope**								0.243P=0.160

For each correlation, Pearson’s correlation coefficient is shown in the first row, P value in the second row.

### Comparisons among aerobic traits

**Figure 3:**
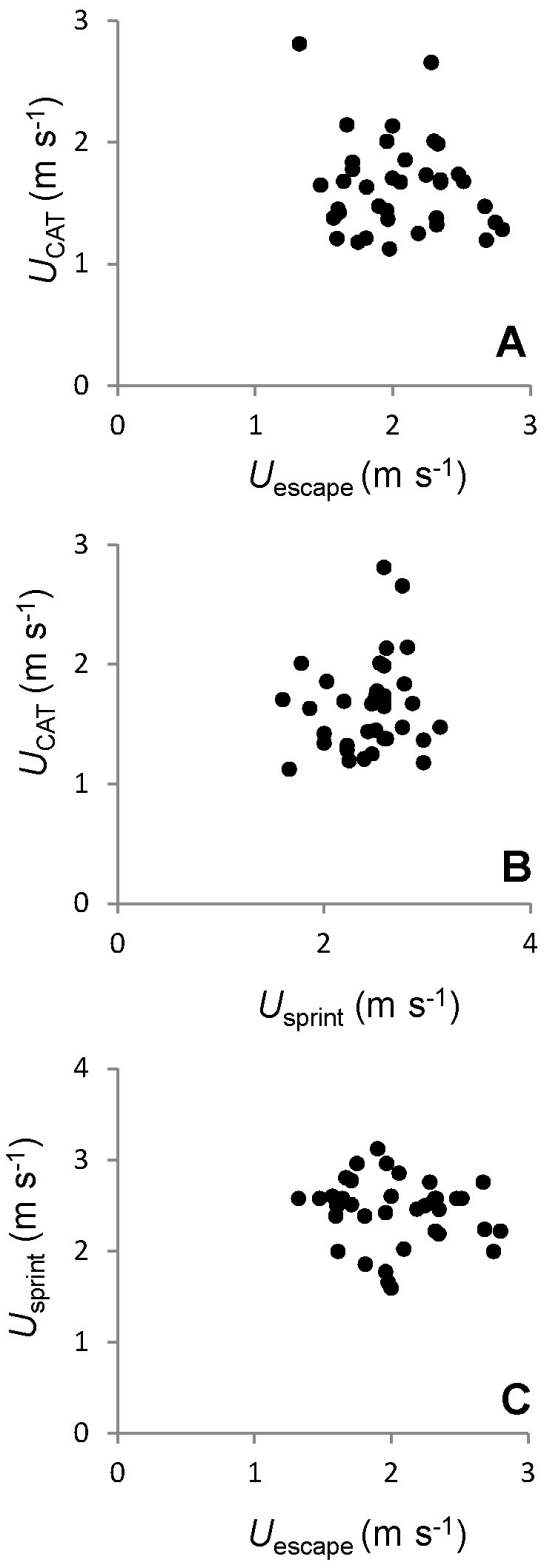
Relationship between RMR and *U*_gt_.

There was no difference between average (0.64 ± 0.08 BL) and maximum stride length (0.65 ± 0.09 BL) measured in individual fish (Paired t-test, P=0.61) and they were highly correlated (Linear regression, P<0.01). As a consequence, maximum stride length only was tested for correlation with the other aerobic traits. There was a positive correlation between maximum stride length and *U*_gt_ (P<0.01, Table 2), however, stride length was not related with any of the other aerobic traits (all P>0.05, Table 2). *U*_gt_ was negatively correlated with RMR (P<0.05, Figure 3, Table 2), but there were no correlations with AMR, aerobic scope and recovery time (all P>0.05, Table 2). Aerobic scope was positively correlated with AMR (P<0.01, Table 2) and negatively correlated with RMR (P<0.05, Table 2), but it was not related to recovery time (P>0.05, Table 2).

### Comparisons among anaerobic traits

**Figure 4:**
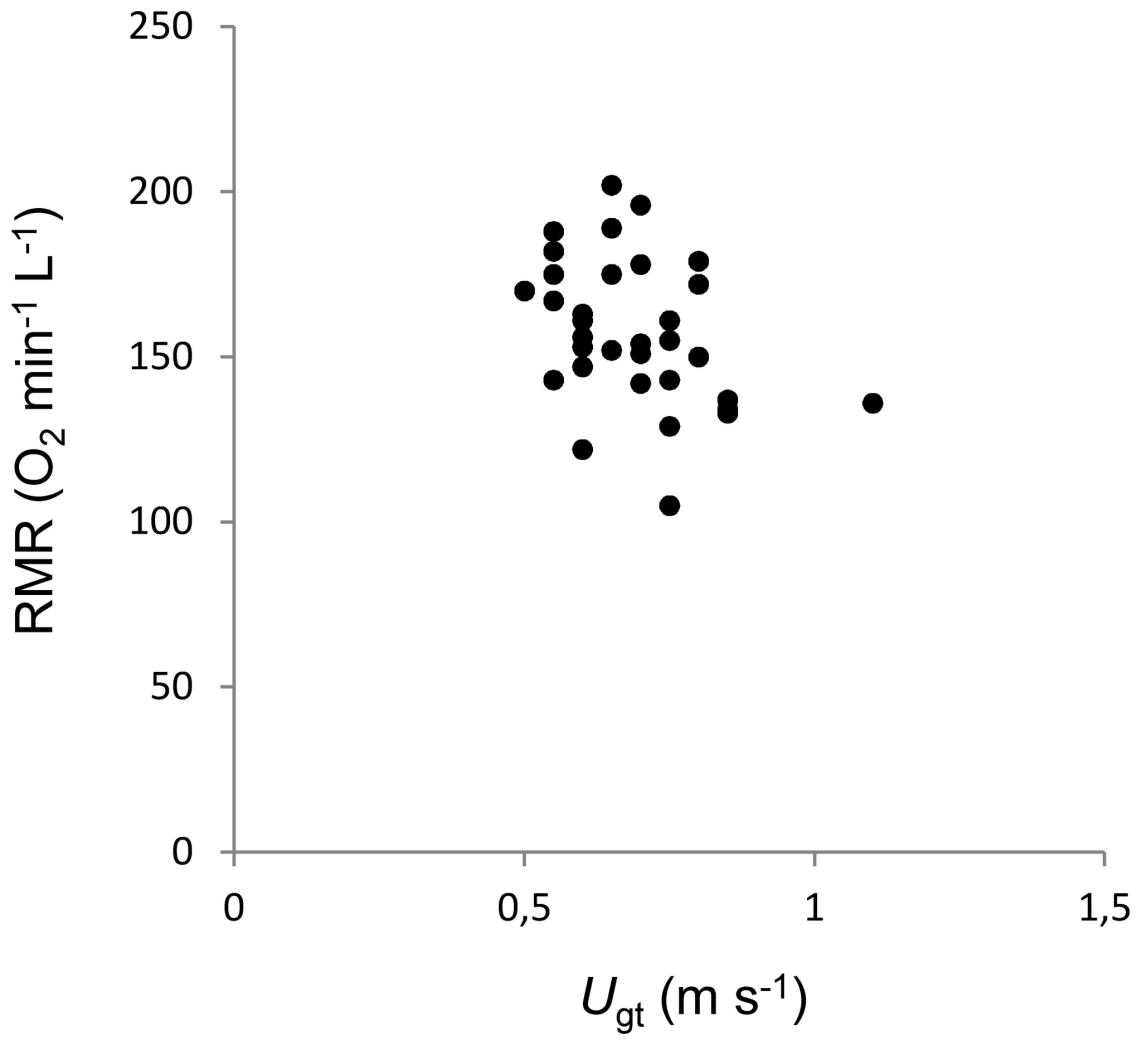
Relationship between (A) *U*_CAT_ and *U*_escape_, (B) *U*_CAT_ and *U*_sprint_ and (C) *U*_sprint_ and *U*_escape_.

Although a positive correlation among anaerobic traits was expected, in particular because they all rely on the use of the same muscular structure, no significant correlations between these traits was found (all p>0.05, Figure 4A,B and C, Table 2).

### Comparisons of maximum aerobic and anaerobic swimming performance

**Figure 5:**
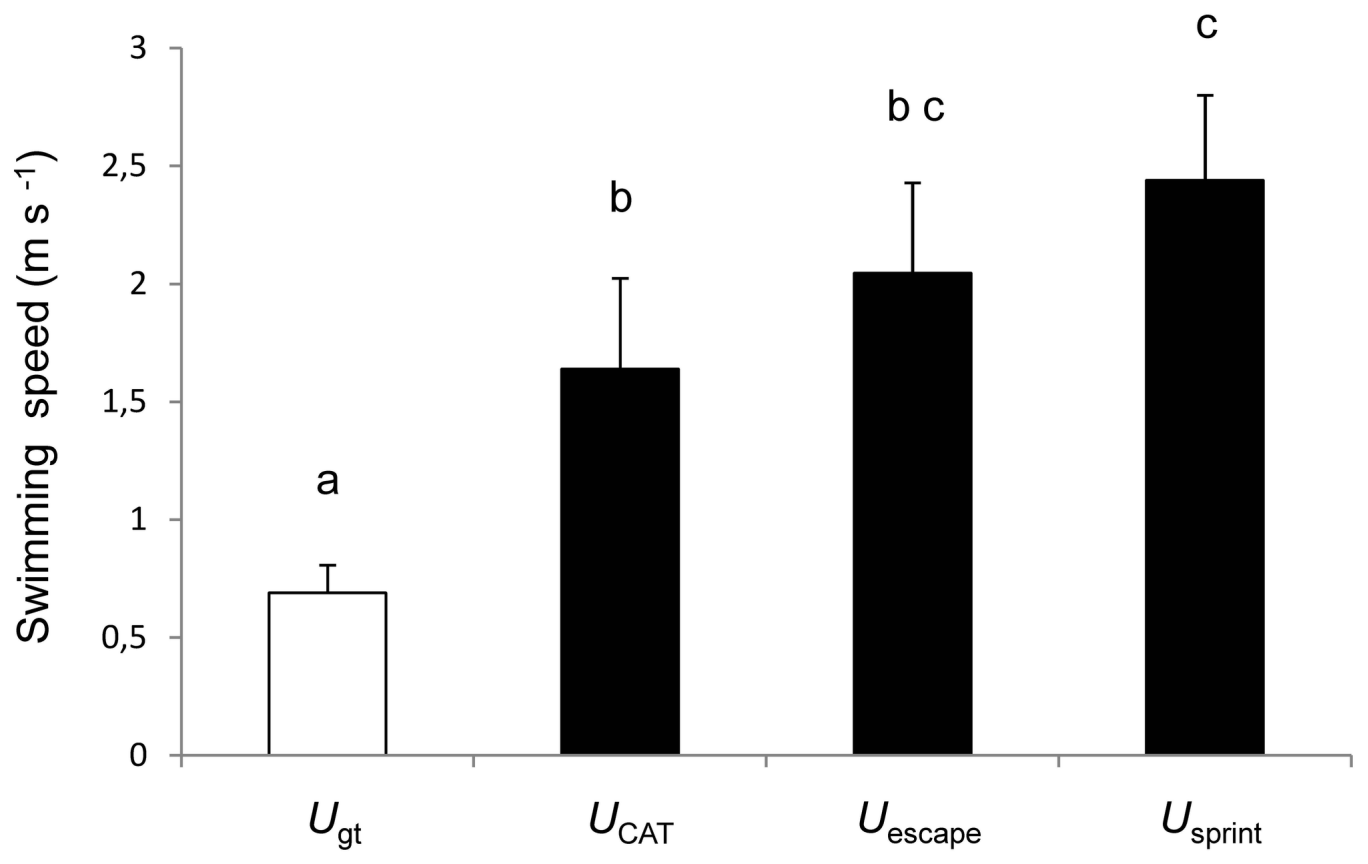
Maximum speeds achieved during the 4 swimming tests. Means not sharing a common superscript are significantly different (ANOVA, P<0.05). Values are mean ± SD.

As expected, aerobic performance (*U*_gt_) was significantly lower than the speeds achieved anaerobically (Figure 5). *U*_sprint_ was significantly higher than *U*_CAT_ (P<0.01). *U*_escape_ was not statistically different from *U*_sprint_ and *U*_CAT_ (P>0.05) (Figure 5). The *U*_CAT_ was the most variable performance trait, with a CV of 23.5%, followed by *U*_escape_ with a CV of 18.7% and *U*_gt_ with a CV of 17.1% (Table 1). Finally, *U*_sprint_ was the least variable of the performance traits, with a CV of 14.8%. Individual variation of *U*_escape_ and *U*_sprint_ was highly repeatable within the experimental protocol (*U*_escape_, ICC=0.79 and; *U*_sprint_, ICC=0.88).

## Discussion

The current study revealed significant variation in all of the performance traits measured. However, the data did not support the hypotheses of a negative correlation between traits that rely on different muscle systems, and we found no evidence of trade-off between aerobic and the anaerobic performance in sea bass. More surprisingly, no positive correlations was observed between the various anaerobic swimming performances although they relied on the same muscle system.

### Critique of methods and comparisons among maximum swimming speeds achieved using the different tests

It is well established that the traits that we measured in this study are repeatable for a given individual, over at least the short to medium term (weeks and months), in teleost fishes [3–5,29]. Thus, whatever underlies variation in performance, it is consistent among individuals. Previous studies have established that our protocols to assess *U*_escape_ and *U*_sprint_ provide repeatable measures over a period of several hours [3,5]. Moreover, although a broad range of techniques were used to describe the various dimension of sea bass swimming performance, obtained mean values for MMR, AS, *U*_gt_, *U*_CAT_, *U*_escape_ and *U*_sprint_ are consistent with those previously published for sea bass of similar mass tested at similar temperatures [3–5,37,41,48]. This coherent and comprehensive data set, combining the various components of sea bass swimming performance and associated elements of bioenergetics, contributes to the value of the present study.

The highest absolute speeds achieved by the sea bass were in the sprint chamber, although *U*_sprint_ were not significantly higher than *U*_escape_. Nonetheless, it is important to note that *U*_sprint_ was significantly higher than *U*_CAT_, thus swim flumes are not reliable for providing measures of maximum anaerobic swimming speeds [49,50]. This assumption is strengthened by the fact that values of *U*_CAT_ have been measured higher than other maximum swimming speeds obtained with different protocols in a swim flume (i.e. *U*_crit_) [51]. One reason for such difference among *U*_sprint_, *U*_escape_ and *U*_CAT_ may be that *U*_sprint_ is measured over less than two seconds and after a period of repose, so presumably endogenous ATP and PCr stores are sufficient in the white muscle fibres, and the test does not exhaust these. The maximum speed measured in a swim tunnel with a CAT is achieved by a constant increase in workload until fatigue, thus the test presumably depletes energy stores in the white muscles once they are engaged beyond *U*_gt_.

### Relationships between aerobic versus anaerobic traits

We found no evidence of a trade-off between maximum aerobic and anaerobic swimming performance in the sea bass. In human athletes, previous work has demonstrated that sprinters have a greater proportion of fast-twitch anaerobic muscle fibres than endurance athletes, and conversely, a greater proportion of slow-twitch aerobic muscle fibres has been measured in endurance runners [52]. It appears that a trade-off in muscle composition might occur that allows a human athlete to excel at either endurance or sprint, but not both. In tetrapods, skeletal muscle comprises a mixture of slow twitch aerobic and fast twitch anaerobic fibers, so increasing the volume of one type of fibre necessarily requires a reduced volume of the other [11]. In fish, although a trade-off has been hypothesized [26,32,53], its absence is consistent with the fact that aerobic and anaerobic muscular structures are anatomically distinct and can work independently [3,33]. For teleost fishes, being good at aerobic swimming may not occur at the expense of anaerobic performance, and *vice versa* [3,30,33], although some work demonstrated that this trade-off may occur in Atlantic cod [10]. The fact that variation in aerobic performance may not always explain variation in anaerobic performance raises the question of why inter-individual variation in these traits persists. The most obvious explanation is that they may trade-off with other morpho-functional traits that we did not measure. The variation in these traits may also persist because they have limited ecological relevance and are under weak selection, if at all in the wild. For example, it is not known how often fishes actually use maximum aerobic swimming performance in the wild [54]. On the other hand, there is evidence that individual variation in escape performance can influence survival of predator attacks [55].

The fact that we found no positive correlations between aerobic and anaerobic performance also contradicts the “good athlete/bad athlete” hypothesis [10]. In general, our results clearly indicate that there is no divergent phenotypic selection pulling individual performance towards different adaptive peaks within the sea bass population that we investigated.

The lack of relationships between anaerobic swimming performance, in particular *U*_CAT_, and traits of aerobic metabolism such as AMR, aerobic scope and recovery time, was unexpected. During anaerobic swimming, the rapid hydrolysis of phosphocreatine (PCr) and the breakdown of glycogen provide most of the ATP in white muscle fibres. Following exercise, ATP and PCr stores may be replenished within 1 h post-exercise [56,57] but re-synthesis of glycogen and recovery from lactate accumulation can require 12 h or more [57,58]. We had expected, therefore, that fish with higher anaerobic capacity (i.e., higher *U*_CAT_) would also have higher aerobic capacity (i.e., shorter recovery from exhaustion) [59]. It has been speculated that teleost white muscle might also have a small aerobic component that is engaged during anaerobic exercise [54,60,61]. It is possible, therefore, that different aerobic contributions to *U*_CAT_ between individuals may have led to the lack of correlation between that performance and recovery time from exhaustion. It is also possible that inter-individual differences in red and white muscle mass can have an effect on recovery from lactate accumulation.

It is known that fish species differ in their ratios of red to white muscle mass, based on their lifestyle [62]. Fish adapted for wide-scale cruising tend to have a large proportion of red muscle, whereas sit-and-wait predators have virtually none [63]. Very little is known about how the relative masses of these muscles might vary among individuals within a species. In the common New Zealand smelt *Retropinna* *retropinna* , there are differences in volumes of each muscle type between sedentary lacustrine and post-migratory riverine populations [64]. However, to our knowledge, nothing is known about variation in muscle type ratio within a given population. Such variation may hide a possible relationship between aerobic and anaerobic swimming performance.

### Relationships among traits of aerobic swimming performance

It was to be expected that stride length and *U*_gt_ were positively correlated because maximum aerobic speed depends on maximum stride length and tail beat frequency at *U*_gt_ [65]. Caution is, however, advised in interpreting our stride length measurements because these should be measured at steady water velocities whereas we measured them during constant acceleration, which can lead to their being underestimated [66].

### Relationships among traits of anaerobic swimming performance

Although *U*_sprint_, *U*_escape_ and *U*_CAT_ rely on the same axial muscle blocks, we found no correlations among these performance traits. There are a number of possible explanations for this. These anaerobic traits differ in their mechanics even though they rely on the same muscle. The fast-start escape response consists of a unilateral axial muscle contraction (stage 1) that quickly propels the fish in a direction away from the threat [28,67,68]. This can be followed by a second contraction powered by the contralateral muscles (stage 2). The escape response is triggered by the Mauthner cells, a pair of large reticulospinal neurons which receive various sensory inputs (visual and mechanoacoustic elements [69]). Sprint performance comprises a number of sequential powerful tail beats, that thrust the fish forward to capture prey or evade predators [29,35]. Beyond *U*_gt_, *U*_CAT_ depends on repeated bursts of white muscle activity, interspaced by coasting, which are used at increasing frequency to meet the constant increase in current velocity in the swim tunnel [4,10]. Although these three types of performance rely on the use of the same muscular system, it is possible that different portions of the muscle, from different parts of the body, are recruited to different extents. It is also possible that other factors, besides individual muscular physiology, such as body form and mass, or caudal fin area, may be important determinants of whole animal performance and thus contribute to variation in the different performance traits.

### Concluding remarks

This study has raised more questions than it has answered regarding the causes and ecological significance of individual variation in aerobic and anaerobic swimming performance in teleost fishes. Given the obvious importance of swimming to the lifestyle of species such as the European sea bass, this therefore remains an important topic for future studies.
